# "I washed and fed my mother before going to school": Understanding the psychosocial well-being of children providing chronic care for adults affected by HIV/AIDS in Western Kenya

**DOI:** 10.1186/1744-8603-5-8

**Published:** 2009-08-23

**Authors:** Morten Skovdal, Vincent O Ogutu

**Affiliations:** 1 Institute of Social Psychology, London School of Economics & Political Sciences, UK; 2 WVP Kenya, Bondo Town, Kenya

## Abstract

With improved accessibility to life-prolonging antiretroviral therapy, the treatment and care requirements of people living with HIV and AIDS resembles that of more established chronic diseases. As an increasing number of people living with HIV and AIDS in Kenya have access to ART, the primary caregivers of poor resource settings, often children, face the challenge of meeting the requirements of rigid ART adherence schedules and frequent relapses. This, and the long-term duty of care, has an impact on the primary caregiver's experience of this highly stigmatised illness – an impact that is often described in relation to psychological deprivation. Reflecting the meanings attached to caregiving by 48 children in Western Kenya, articulated in writing, through photography and drawing, individual and group interviews, this paper presents three case studies of young caregiving. Although all the children involved in the study coped with their circumstances, some better than others, we found that the meanings they attach to their circumstances impact on how well they cope. Our findings suggest that only a minority of young caregivers attach either positive or negative meanings to their circumstances, whilst the majority attaches a mix of positive and negative meanings depending on the context they are referring to. Through a continuum of psychosocial coping, we conclude that to provide appropriate care for young carers, health professionals must align their understanding and responses to the psychosocial cost of chronic care, to a more nuanced and contextual understanding of children's social agency and the social and symbolic resources evident in many African communities.

## Introduction

*"My caring experiences make me feel happy, they will help me in the future. If I am left alone, I will be able to do the duties. I become strong; I don't become a weak child." *Joyce, age 12

In a globalised world, the management and support of people living with AIDS is an issue that concerns us all [1]. With our growing understanding of HIV and AIDS and improved access to antiretroviral therapy (ART), the AIDS epidemic is amenable to intervention and treatment management, changing its course to mirror the disease processes of other chronic illnesses [2]. According to Thorne [3] chronic illnesses are long term, and require careful management and adjustment by the patients and their caregivers as the person with the disease may fluctuate between chronic and acute episodes. People living with HIV/AIDS also require careful management to sustain their health. This is particularly the case of those on ART whose diet, adherence to rigid treatment plans and psychosocial well-being has to be managed, a responsibility that is often shared with their primary caregiver.

Although ART coverage in sub-Saharan Africa increased by 33% in 2007, with 2.1 million people receiving ART [4], resulting in a decline of HIV/AIDS related mortality [5], numerous external factors impact on the success of ART, particularly in rural Africa [6]. It is therefore important to understand the action and decision processes that impact on ART adherence [7] and home-based care of people with HIV/AIDS [8]. Poor infrastructure and opportunity costs have meant that many people still go without life-improving medicines [9] and many of those who receive ART are unable to adequately adhere to the strict treatment plans [10,11]. A study in Tanzania reveals that fear of stigma and discrimination, additional costs of transportation, and supplementary food and negative associations with hospital staff, have deterred HIV infected people from following up on referrals for ART [12]. Non-adherence can result in relapses and drug resistance, and recent findings from Malawi suggest that poor compliance to ART can even result in increased mortality [13].

These difficulties highlight the importance of understanding the implications for long-term care of people living with AIDS. Primary caregivers are at the forefront, struggling with rigid ART adherence schedules and frequent relapses. Nevertheless, primary caregivers continue to play a crucial role in facilitating the adherence of treatment plans and the provision of economic, nursing and moral support [8,14]. Perhaps unsurprisingly, family caregiving is often perceived to be a burden on the caregivers and they are increasingly reported to be at risk of poor health and deprived psychosocial well-being [15,16]. The majority of research on informal caregivers of people living with AIDS is focused on women [8,17], however, growing attention is being given to young caregivers. In this article we use the term 'young carers' to refer to children under the age of 18 who provide nursing care and support for sick, disabled or elderly relatives or guardians affected by AIDS on a regular basis and play a key role in sustaining the household.

Research on young carers in Africa is still in its early stages and has so far been limited to the context of AIDS. Elsbeth Robson and colleagues have been in the forefront, identifying the circumstances that characterise young caregiving in Africa. They have explored their caring arrangements [18], their duties and responsibilities and how this negatively impact on their school attendance [19], the socioeconomic and structural influences that induce young caregiving in Africa [20,21] as well as the ethical implications of doing research with young carers [22].

Robson and colleagues have been cautious not to export western conceptualisations of young caregiving to the African context [18], and have highlighted the reported benefits of young caregiving [19]. Evans and Becker [23] in their recent comparative study of children caring for parents with HIV and AIDS in Tanzania and UK, approached children as social actors and usefully identified some of the social determinants that facilitate the resilience of caregiving children. Nevertheless, the needs and vulnerabilities that do characterise young carers, together with a predominant focus on the ill-effects of caregiving in the international literature on young carers [24,25], and emerging trends on exploring the psychological distress of children affected by AIDS [26-33], has encouraged a focus on the psychological well-being of young carers, usually starting with the assumption that caring is inherently a source of mental ill-health for young people.

As with their adult counterparts, children who provide care and have domestic responsibilities have been associated with fragile mental health [16,22,34-39]. In a comparative study between Zimbabwe and the United States, Bauman and colleagues [34,35] interviewed a total of 100 ill mothers living with HIV and AIDS and one of their children. By choosing to use depression scales, the study begins with the assumption that caring for an ill parent is an inherently traumatic experience that automatically puts children at risk of mental health problems. While this undoubtedly is true in some cases, we believe that this assumption reflects a Western mental health discourse and dominant representations of childhood as a period of innocence and mental fragility in the absence of adult protection.

Although a debate on the psychosocial well-being of young carers is imperative, we believe a different approach is required to provide meaningful psychosocial support to young carers. This approach can lead to a more profound understanding and knowledge of coping and well-being and provide us with a good starting point for moving toward better health. Such an approach has been usefully theorised by Antonovsky [40] who, through his theories of salutogenesis (latin for the origins of health) and 'sense of coherence', argues that the meanings given by people in difficult circumstances to their life situations shapes their sense of coherence, which then impacts on how they cope with their circumstances [40,41]. In taking a salutogenic approach we are not seeking to develop specialised techniques or suggestions about how outside professionals can cure the stress and hardship faced by young carers through expert techniques such as psychotherapy. Rather, we seek to report on existing indigenous life strategies – developed by young carers themselves within their immediate communities – that facilitate the sense of coherence they construct for their lives and promote their movement toward coping and well-being. Meanings ascribed to stressful life events have previously shown to be critical in coping and promoting psychosocial well-being [42,43].

In line with Antonovsky's salutogenic approach and to broaden our understandings of the psychosocial well-being of young caregiving, this paper presents some of the characteristics that exemplify the circumstances of young caregivers, including the social resources available to them, and the meanings that they attach to caring. In doing so, the paper aims to outline their negotiation with local understandings of childhood as a time of duty and service through their cognitive ability to attach a meaning to their caring experiences and construct a positive identity around these meanings.

We began this paper by quoting a 12-year-old girl who spoke of her caring role as a source of strength, even happiness. This counterintuitive claim calls for an understanding of the psychosocial nature of the demands of child caregiving. In this paper we therefore seek to explore the psychosocial well-being of children providing care for people chronically ill from AIDS, suggesting that the psychosocial well-being of young carers is best promoted with a nuanced understanding of the circumstances that surround young caregiving.

## Methodology

This paper reports on the first phase of a two-year action research project with young caregivers in Western Kenya. This qualitative study was granted clearance from the LSE ethics committee and the Department for Gender and Social Services in Kenya.

### Setting

The project is located in Bondo district along the shores of Lake Victoria. Poverty is a major challenge for people in Bondo district. With 68.1% of the 261,000 people living in the district living in absolute poverty, Bondo is one of the poorest districts in Kenya [44]. Bondo also has one of the highest HIV prevalence rates in Kenya. Estimates from the 2002 district development plan [45] put the HIV prevalence rate in Bondo at 30% while a more recent and conservative figure estimates it to be 13.7%, still double the national average [46]. Until 1999, the Bondo area was part of the Bondo-Siaya district. Throughout the early stages of the HIV and AIDS epidemic, it was marginalised due to the poor reach of health services that concentrated in the Siaya area, presumably contributing to its exceptionally high HIV prevalence rates. Another explanation for high HIV prevalence rates is found in its geographical location. Bordering Lake Victoria, the fishing villages provide employment for fishermen and truck drivers from all over Kenya whose migration and constant movement have contributed to the spread of HIV and AIDS. Numerous international NGOs have since been established in the Bondo area in an effort to halt the spread of AIDS and seeking to promote home based care and orphan care and support. With ARVs freely available in Kenya, an increasing number of people have commenced antiretroviral therapy. Currently, an estimated 40% of those infected with HIV and AIDS in Kenya receive ART [4,47]. Whilst this positive development also holds true in Bondo, the opportunity costs and poverty characterising the district undermine the effectiveness of ART. A combination of the distance and costs of travelling to the nearest ARV health facility, stigma, and an inadequate diet all contribute to frequent relapses and the continued need for care. Relapses, and the fact that 60% of people living with HIV and AIDS are still without ART [4] mean that many children in Bondo district have had their lives affected by AIDS, often providing chronic care and support to those affected by the disease. The two participating rural communities in Bondo district are situated in areas characterised by high HIV prevalence rates and research from a neighbouring division suggests that one out of three children below 18 years of age have lost at least one biological parent, and one out of nine have lost both biological parents [48]. Although Kenya has a relatively low national HIV and AIDS prevalence rate (6.7%) compared to other Southern African countries, we believe Bondo district is representative of many rural areas in some of the hardest hit countries in sub-Saharan Africa.

### Data collection and Analysis

In this paper we present three case studies in order to map out the types of experiences reported by children, and to locate them on what we will call a 'continuum of coping'. These case studies represent the end point of a lengthy and stage-wise process of data collection and analysis that we will outline in this section.

Our data collection involved photography, individual and group interviews involving 48 young caregivers aged 11 to 17. Approaching the children as experts on their own lives [49], multiple methods were used to gather the data to ensure all children had an opportunity to communicate about their experiences in a way that felt comfortable [50]. Adapting the photovoice methodology and process developed by Wang and colleagues [51-53], the generation of photovoice data involved four stages. The first stage was that of photo-taking. Over a two-week period children took photos guided by the following four questions: 1) 'What is your life like?', 2) 'What is good about your life?', 3) 'What makes you strong?' and 4) 'What needs to change?' The second stage involved getting children to choose six of their favourite photographs, encouraged to identify a mix of photos showing how they get by, things they lack and/or something that is important to them. In the third stage, the children reflected on their chosen photographs and wrote down their thoughts prompted by the following questions: 1) 'I want to share this photo because...', 2) 'What's the real story this photo tells?', and 3) 'How does this story relate to your life and/or the lives of people in your neighbourhood?' If the children wanted to share a story that they were unable to capture on camera (e.g. for ethical or practical reasons), they were encouraged to draw the situation. This exercise generated a total of 184 photos and 56 drawings, each accompanied by a written reflection/story. To further explore the findings generated from their written reflections, 24 individual interviews and two group discussions were conducted with the children.

**Figure 1:**
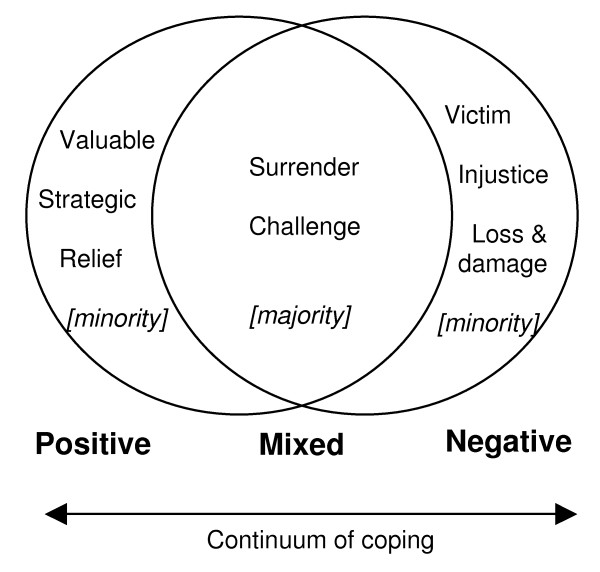
**Diagram of Meanings attached to caring**.

This paper is part of a much wider study, which yielded, amongst other things, a 6 theme analysis of key themes structuring children's accounts of their experience: 1) dynamics and characteristics of luo society; 2) characteristics and perceptions of caring children in Western Kenya; 3) determinants of caring experiences; 4) social resources; 5) action-based coping and 6) psychology-based coping [cf. [54]]. These themes cut across individual accounts and reflect general representational resources identified across the 48 research participants. In this particular paper we focus on three individual life stories, presenting the stories of three children in ways that highlight the different ways in which children used these representational resources to give meaning to their lives, and how different life experiences/access to resources and supportled to varyingly positive, negative or mixed evaluations of their caring experiences (see Figure 1).

We have also taken the liberty of abridging some of the more detailed narratives into poems, which summarise the content of their accounts of their lives in a way that transcends the narrative chronology whilst bringing forward their meanings, using the children's own words [55,56].

## Findings

All children managed their caring responsibilities and coped despite adverse circumstances. However, different meanings attached to caring suggest that the psychosocial cost of caregiving should be viewed on a continuum of coping. As diagram 1 indicates, most of the children surrender to the circumstances prescribed to them and 'get on' with the challenge of providing care. They may attach both positive and negative meanings to their experiences depending on the context and circumstances. However, a minority of children saw caring either as a relief, something valuable they would not be without, or as something which has caused damage to their life. Through the case studies of Samuel, Carolyne and Pascal (pseudonyms have been used to protect the identity of the children) we present three different scenarios of life as a young caregiver and how these circumstances may contribute to the meanings they attach to their circumstances.

The meanings that young carers attach to their circumstances are influenced by the social environment. In particular local understandings of childhood were found to impact their sense of coherence. Carolyne, during an interview, explained that "the duty of a child is to help parents. A child is called a helper." Concurrent to the local perception of childhood as a time of duty and service is a sometimes conflicting understanding of childhood, which is more rights-based and stresses the importance of education. It is common for children and adults in Bondo to draw on both representations of childhood, depending on the context, and they should therefore not be seen as binary, but located on a dynamic continuum. Nevertheless, as the case studies will illustrate, it is against these understandings of childhood that young carers give meaning to their circumstances as they manage their caregiving, head-of-household duties and education.

## Samuel, age 13 (positive meaning)

Samuel was 9 years old when he first realised that his father was ill. He noticed his father's swollen hands and joints and explained this in terms of a spell having been cast on his father by someone who was jealous of his job. Soon after, "another spell was cast by another person on his legs" and he was bedridden. Although he was taken for prayers, his condition never changed. He had sores all over the body and Samuel applied creams to his body, washed him, gave him drugs, prepared food and spoon-fed him. Although his mother was around to help, she too got sick and was able to offer less and less support. Samuel's father died in 2005 when Samuel was 11 years old and he currently cares for his mother. Samuel is not caring for his mother alone. His little sister also helps by fetching firewood and water, and assists Samuel where ever she can. Samuel also has a supportive extended family network. His aunt has moved in to help them out. Aware of this support, Samuel's assessment of his situation allows him to reflect further on local understandings of childhood as a time of duty and service and his perceived identity and role as a young carer. These reflections led him to move in with his ageing neighbour who had been deserted by his family: "I went and lived with him, helping him out, cooked for him, fetched water, took care of the poultry and ensured he was clean." Rather than concentrating on the care of his mother, Samuel's decision to move in with his ageing neighbour not only reflects his commitment to be a young carer but also his assessment of his circumstances as low risk.

Samuel has always played an important role in sustaining the households in which he has lived, drawing on the resources available to him through his parents. He cultivates land with sorghum, some of which he sells to buy basic amenities or chicken or goats. "Chicken help me in providing eggs, meat and gives us money if we sell them [...] at times I sell a chicken to get school fees". Samuel's family also has a cow whose milk can pay for his school uniform and which can be slaughtered for a funeral. The resources available to Samuel, combined with his active participation, allows Samuel to cope. From a psychosocial perspective, the availability of social resources and support makes it easier for Samuel to actively identify himself with childhood as a period of duty and service, successfully exceeding local expectations of childhood and from that create a positive caring identity. This positive identity came out strongly in his narratives. Summarising his narratives, the poem below indicates that both his religious faith and his mobilisation of local understandings of childhood have enabled Samuel to create a positive caregiver identity, based on the acceptance, love and blessings he gets in return for his caring, both from the community and God. Samuel therefore sees caregiving as a strategy through which he derives recognition and support from the community. In addition, Samuel distinguishes himself from other children, arguing that "if I have something I can share it with other children, I don't deny them." Samuel values this quality highly and attributes this good quality to his caregiving experiences. He believes that many other children "don't share what they have with others, they pretend not to have anything." The poem summarises and links the positive meanings that Samuel attaches to caregiving and the construction of a positive caregiving identity, one that influences Samuels psychosocial well-being and coping.

I HAVE A HELPING HEART

How are you different?

I like helping people.

I have a helping heart,

if I have something I share it

What makes you happy?

All that I have done makes me happy

the villagers love me seriously

as I don't do bad things in the community

How is life as a carer?

If I care for a sick person,

I'm happy since I get blessings from God

It is not good for one to suffer.

There are no negative effects

## Carolyne, age 15 (mixed meanings)

Carolyne's father died of an illness when she was 7 years old and she began taking care of her mother at the age of 10. For two years Carolyne provided nursing care and psychosocial support to her mother and kept the household running. She cooked and fed her, washed and massaged her body. In addition to the nursing care, "I also did the other house work such as cleaning the house, washing utensils and fetching water". The workload was heavy and interrupted with a change when her mother was admitted to the hospital. In the absence of adequate nursing care at the hospital, Carolyne continued to care for her mother in the hospital, forcing her to leave school. Carolyne's mother died in the hospital. During this time, she was largely coping by herself – possibly as a result of the stigma associated with AIDS. But her caring experiences do not end here.

Carolyne and her little sister then moved in with her grandmother who was very old and required care and support. All her children had died and she was dependent on her grandchildren. Despite taking on significant caring and household chores, Carolyne was determined to return to school. But soon after re-entering, her class teacher advised her to leave school as she was not attending classes consistently due to her responsibilities at home. In addition to her poor attendance, Carolyne suffered from slight visual impairment and had difficulties reading what was written on the blackboard. Determined to stay in school, Carolyne refused to drop out and continued with her education. Whilst dealing with her personal health issues and education, Carolyne also lost the support of her little sister: "My sister was sponsored [to go to school] by some whites and she is now in Nakuru". Aside from her sister, Carolyne does not mention receiving support in her provision of care from anyone else. This is also reflected by the amount of time she reported to spend on caring, "you work throughout the day, no time for resting". She did however occasionally negotiate her way to material or food support from community members. In describing a photo of a woman who has supported her, she says". This photo reminds me of the kind of support and love we get from the community members. If I need anything, I tell them and if it is available, I will get it".

After two years of caring, Carolyne's grandmother passed away. She was left to stay with her grandfather who was mistreating her, often leaving her to spend nights outside where she describes her encounters with hyenas. Carolyne quickly moved in with her aunt, who has been supporting her well since, "If I ask my aunt to buy me something she will not ignore me, even though I am not her child. No, she will do for me the same as for her own children". Carolyne and her aunt jointly provide care for her aunt's elderly co-wife.

Like Samuel, the numerous caring experiences that have confronted Carolyne have facilitated a caring identity. Unlike Samuel, her identity is a more reflective one, acknowledging both the negative and positive impact of caregiving. Although she is now in foster care with her aunt (whom she calls mother), she continues to provide care and support for the sick and old in her community, this time with help from her aunt. Although Carolyne says that "the lives of children caring for the sick is not good", she describes her circumstances and work as something she just had to do, without complaints, and something which is important. Carolyne's experience highlights both the difficulties she has faced in providing care, as well as the way she has dealt with them. Aside from the periodic support she has received from her sister and aunt, her social environment has been of limited support, yet she accepted her role as carer and simply 'got on' with the job.

As the poem summarising Carolyne's accounts demonstrate, Carolyne has mixed feelings toward her circumstances, acknowledging both the negative and positive impact caregiving has on her, consciously accepting her circumstances.

All that I have done has been important

I was first caring for my mother

when she was sick.

She was too sick to do anything.

I was the one to wash her and feed her.

It reached a point where I could not sleep.

She was crying of pain all the time.

She needed water and wanted to be massaged.

I also did the housework.

Cleaned the house, fetched water and prepared food.

I had to leave school.

After her death my grandmother fell sick.

I started caring for her.

We had no money, I could not take her to the hospital

I have had problems.

I have been committed to caring and had little rest.

But all that I have done has been important.

## Pascal, age 14 (negative meaning)

Pascal began providing care from a very young age. Until his seventh birthday, Pascal was taking care of his father who suffered from AIDS. Pascal did not receive much support from his mother, who eventually left the house because of stigma, leaving 6-year-old Pascal alone with his father. Pascal's older siblings lived away and only provided limited support, mostly in terms of food. Pascal spoon fed his father and cleaned his body, also in the most intimate of places. When Pascal tried to seek out help from his mother who was staying with her brothers, he was chased away by his uncles. When asked about how he coped with the situation, Pascal explained: "I had a vegetable garden which I used to cultivate and sell the produce from in order to buy drugs for my father or anything else he needed". At the age of seven, Pascal's father died. Following his father's death, Pascal moved in with one of his brothers and returned to school. To Pascal's dismay, he was told by his teacher that he had to repeat class 2. Pascal had a difficult year: "It was painful to see my classmates in class 3. I did not forget the caring of my father for the whole first term and I could only think about how my father died. This thinking left me at the bottom of my class. But in term 2 I started to forget these things slowly". As with Pascal, many young carers attach a feeling of loss or damage to their education as a result of time consuming caregiving or a lack of concentration.

Pascal's caring experiences did not end here. A couple of years later his brother also got ill, but this time Pascal was not alone in caring: "Fortunately we were two of us, so one cared at night and another during the day. I especially cared during the night and it made me unable to concentrate as I almost slept in class". As his brother got bedridden, Pascal left school once again and had to repeat class four when his brother died. Following the death of his brother, Pascal moved in with his grandmother where he currently stays. He supports his grandmother with those tasks she cannot perform due to old age and is reviving his relationship with his mother, who decided to return to the community two years after his father's death. In reflecting on the limited support he received during his caregiving, which was a key contributor to the negative meanings he attach his experiences, Pascal laid heavy emphasis on the relative poverty that he endured: "Other children have their school fees paid for by their parents, they have good clothes, good shoes and they look nice whilst I cannot afford to look nice because of the little money I have". In comparing his life with other children, Pascal clearly sees himself as a victim and feels the injustice surrounding his circumstances. Despite these victimological representations, Pascal has not lost hope: "I know and hope that my life will be good".

The poem summarising Pascal's experience exemplifies the very difficult conditions in which Pascal was providing care. It is evident from Pascal's narratives that he feels a tremendous sense of loss and damage to his life, circumstances that have pushed Pascal to feel a sense of relief following the death of his father.

All this suffering

**Figure 2:**
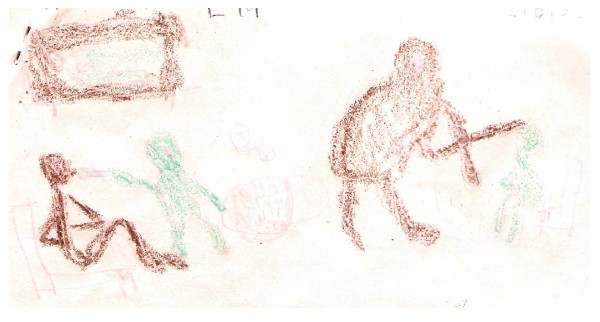
**Drawing by Pascal**.

This drawing (see figure 2) shows the kind of care I have given to the sick

My mother was nowhere to be found

My father's sickness got worse and worse

It forced me to leave school

He was unable to walk

I washed off his faeces

He disturbed me during the night

I was very sad, I was left alone with my father

When he died, I thanked God, he made me suffer a lot

One of my brothers fell sick, this also made me leave school

I had to repeat class four when my brother died

All this suffering made me go to my grandmother's place

I am now in class 7 and learning

I help my grandmother with harvesting,

fetching water and cooking

It shows good behaviour and the majority loves me.

I am still not happy with the kind of life I am living,

though I am in school

## Discussion

Our three case studies highlight the duties involved with chronic care of adults affected by HIV/AIDS. In the absence of working adults in their households, many of the children were responsible for the generation of food and income to sustain their household. Alongside these responsibilities, children showed great concern for the care and well-being of their sick parents or ageing grandparents, taking on considerable nursing duties. Children are often the primary caregivers for their ailing parents and play a fundamental role in the management of their parent's disease and opportunistic infections. Approaching young carers as social actors, the case studies illustrate how young carers cope with the care of guardians who suffered from chronic conditions. We have highlighted the social resources and protective factors potentially available to them (or not) and how these social resources may contribute to the way in which they assess their risk and vulnerabilities.

Samuel received support from his aunt and little sister and used his faith and local representations of childhood to construct a positive identity as a young caregiver. Samuel suggests he is loved by the community because he provides care and support, a reflection of the local expectation of childhood as a period of duty and service. Carolyne, on the other hand, did recognise the disadvantages and the problems she was facing, but also managed to attach some positive meanings to her experiences. She surrendered to her circumstances and got on with caring, approaching caregiving as a challenge, something difficult, but something which was important. Although Carolyne sees caregiving overall as positive, there were other children, able to reflect on a mix of positive and negative meanings, who perceived caregiving as an overall negative thing. This was often upon reflection on the impact caregiving has had on their education. We found only a few children with completely ambivalent meanings. Pascal was much less optimistic about his circumstances, reflecting on his particular situation and lack of social support. Pascal was further influenced by representations of the importance of education, and as caring compromised his education in certain periods, he felt a sense of loss and damage to his chances in life. As is evident from the case studies, their specific circumstances, social resources and representations impact on the meanings which the children attached to caring. This in turn contributes to their psychosocial well-being, a relationship (between ascribed meaning and well-being/coping) that has been documented in the context of physical illnesses [42,57,58].

Most of the children involved in the wider study attached a variety of meanings to caregiving and many were still negotiating the different meanings they attached to various contexts. These mixed meanings were often a reflection of the paradoxical expectations of children. On one hand the children draw on local understandings of childhood as a time of duty and service, and on the other, children are told about the importance of attending school. As young carers having to juggle with both duty and service and education, the meanings they ascribe to their circumstances are often a by-product of their negotiation between these expectations. Nevertheless, the negotiation of meanings that these children actively engage in facilitates their psychosocial coping and connects them to different meanings at different times in order to achieve particular goals.

Whilst certain caregiving circumstances may well be detrimental to a child's mental health, this is not always the case. Counter balancing (or not) some of their vulnerabilities were the symbolic and socioeconomic resources that arose from their familial and social environments, allowing many of the children to draw on some of the more positive aspects of caregiving. The ability of caregiving children to identify and draw on the benefits of their difficult circumstances has previously been identified as a strategy for psychological survival [19]. The three case studies presented in this paper also suggest that the children's previous experiences of caregiving and whom they currently care for is an important influence on the meanings they attach. Carolyne for example, who has provided care and support to a number of adults over a period of time, is now used to caregiving and has a break from the emotional cost of caring for an ailing parent. Carolyne is able to reflect on the hard times she had, but also on the skills and social benefits caregiving has brought her. Carolyne was not an exception. Many of the participating young carers have had numerous caregiving experiences and continue to provide care long after parental death, as their grandparents, who have lost children to AIDS, are in need of care and support as they age. As exemplified by Carolyne, the majority of children, depending on the context and time, approach and describe their circumstances differently, and effectively move around within the continuum of psychosocial coping. Some children have actively constructed a positive caregiving identify, whilst others see themselves as marginalised. These meanings and identities may change with time. These findings suggest that the vulnerability of young carers is a process [59], one which requires a nuanced understanding of the protective factors and social resources available for the children to actively draw [54].

One important finding is that the children are able to identify numerous benefits to young caregiving and actively draw on these benefits to facilitate psychosocial coping. We hope that our analysis has shown that to provide appropriate support for young caregivers, health professionals must align their understanding and responses to the psychosocial cost of chronic care, to a more nuanced and contextual understanding of social agency and opportunities evident in African communities. Rather than focusing on counselling services, interventions targeting young carers should strengthen the existing social resources within their context and provide the communities with the financial and social psychological resources to do so.

## Competing interests

The authors declare that they have no competing interests.

## Authors' contributions

The study was conceived and coordinated by MS. Both authors contributed to the conception and design of the study. VO participated in conducting the interviews and workshops from which the data were collected as well as transcribing and translating the data. MS drafted the paper. Both authors read and approved the final manuscript.
